# The Effect of the Reading Circle Method on Curiosity and Exploration, Creative Reading and Visual Literacy

**DOI:** 10.3390/jintelligence13070074

**Published:** 2025-06-23

**Authors:** Yasemin Baki

**Affiliations:** Department of Turkish and Social Sciences, Faculty of Education, Recep Tayyip Erdoğan University, 53100 Rize, Turkey; yasemin.baki@erdogan.edu.tr

**Keywords:** reading circle, creative reading skills, exploration and discovery visual literacy competencies

## Abstract

This study examined the effects of the reading circle method on the curiosity and discovery perceptions, creative reading skills and visual literacy competencies of Turkish teacher candidates. The study group of the study conducted for this purpose consists of 48 teacher candidates studying in the Turkish language teaching department of a university in the north of Türkiye. A sequential mixed design was used in this study, which was conducted with mixed methods. In the quantitative dimension of this study, a pre-test and post-test, control-group-free experimental design was used, while in the qualitative dimension, a case study design was used. The Life Skills Scale, Individual Innovation Scale and semi-structured interview form were used to collect the data in this study. To examine the effect of the experimental process in this study, the data obtained were analyzed with a t-test for dependent groups; the data obtained from the interviews were analyzed with content analysis. According to the results obtained from this study, the reading circle method significantly affected the curiosity and discovery perceptions of teacher candidates. It was determined that creative reading skills have a significant effect on the sum of the scales and all other subdimensions except for the dimension of interpreting the text and the dimension of giving importance to visuality and interpreting visuals using Office software in visual literacy competencies.

## 1. Introduction

The reading circle method was first introduced in 1982 by Karen Smith when the book club application was adapted to the school environment, and the name of this method was first given by Kathy Short and Gloria Kaufman in 1984 (Tosun 2018). Developed to improve reading skills and provide in-depth comprehension of what is read (Levy 2019), this method is a versatile and high-level reading method that offers individual and group reading opportunities and includes many reading techniques and strategies (Amalia 2025; Briggs 2010; Mohamed 2023).

The reading circle, a student-centered reading method, is shaped according to constructivism theory (reader-response theory, schema theory) and socio-cultural theory (McElvain 2010; Schoonmaker 2014). In this method, which offers the opportunity to experience a multifaceted reading process around these theories, groups formed by the readers’ choices are provided with the opportunity to first read individually a text selected by the readers and then discuss it in groups (Cloonan et al. 2020). Thus, it offers readers a collaborative reading experience that combines individual and group reading (Tracey and Morrow 2006).

Reading circles, which are accepted as an effective reading tool in the education system today, enrich and shape personal learning experiences by increasing interpersonal interaction with this group-oriented perspective (Goa and Wodai 2022; Widodo 2016). This interactive experiential process should be structured according to certain principles regardless of the age group or applied environment. The principles that should be followed in the application process of the reading circle method are as follows (Moeller and Moeller 2007; Daniels 2002):

1. Students choose the materials they will read.
2. Small discussion groups are formed according to the book selection.
3. Group roles should be changed constantly.
4. Different groups read different books.
5. Groups meet regularly on specified dates.
6. Students take notes to guide their reading and discussion.
7. Discussion topics are determined by students.
8. Open-ended, natural discussion is aimed at group meetings.
9. The teacher’s role is guidance.
10. Teacher observation and self-assessment are used together in evaluation.
11. Fun should be part of the application.
12. When the books are finished, each group shares the books they have read with the class through presentations, dramas and other projects.
13. New groups are formed according to new books, and the cycle continues.

In reading circles, the main element that provides different perspectives, creates a suitable discussion environment and forms the basis of this method is the set of roles that students prefer in their reading experience (Tomlinson and Strickland 2005). These roles are divided into two types: basic and optional roles (Daniels 2002). The basic roles are connection maker, questioner, section expert and painter. The optional roles are summarizer, word hunter, action scout, character master and observer. In each circle, the distribution of roles is essential. Group members should each have a role during the discussion; the aim of these roles is to examine and analyze the reading material according to the role during the individual reading process. In accordance with the roles determined before the discussion process, carried out as a group within the framework of the reading circle principles, each group member participates in the discussion according to the role they have undertaken and shares the information they have acquired in this role axis with their group members (Chung and Lee 2012). This system, which puts the individual at the center, offers readers new perspectives with constantly changing roles while also providing the opportunity to recognize the different perspectives of these roles through a collaborative learning experience in group discussions (Calzada 2013; Tracey and Morrow 2006).

In this method, readers actively participate in the reading process and are able to think deeply about the text, discuss it and examine it from an analytical perspective. In group discussions, readers exchange ideas on the text, and their interpretations of the text provide an opportunity to others to gain different perspectives (McElvain 2010). At the same time, the social interaction created by these group discussions, which are structured within the framework of equality, respect and active participation, allows everyone to share their ideas freely and interpret the evaluated text from different perspectives (Morris and Perlenfein 2002). In this way, it both supports individual learning and provides the opportunity to gain collective knowledge by allowing readers to contribute to the creation of a collective meaning by using group dynamics and bringing together different interpretations (Hathaway 2011).

Reading circles are not only a reading method but also an effective reading platform that develops individuals’ social and emotional dimensions and offers them the opportunity to express themselves and participate. It is often used by educators to deepen the process of understanding the text and is also applied in terms of acquiring new strategies (Certo et al. 2010). These reading activities, which are performed in groups, allow for the establishment of connections between readers and the strengthening of relationships between them while also turning this process into an enjoyable and educational experience (Brownlie 2005). In this method, readers are given the opportunity to express themselves, listen to each other respectfully, understand each other and develop a social consciousness. Thus, it contributes to both personal and social development (Calzada 2013).

The literature studies on the reading circle method, which is one of the effective methods that enable the reading process to be carried out consciously, have mainly focused on the development of reading habits, reading interest, reading motivation, reading comprehension strategies, reading comprehension skills and vocabulary (Amalia 2025; Avcı et al. 2013; Avcı and Yüksel 2011; Balantekin and Pilav 2017; Briggs 2010; Camp 2006; Çağ 2024; Goa and Wodai 2022; Karatay 2015, 2017; Tosun 2018; Küçük 2024; Mete 2020; Monaghan 2016; Mrak 2021; Novasyari 2021; Nurhadi 2017; Olsen 2007; Qie et al. 2023; Pitton 2005; Rahman 2022; Rutherford et al. 2009; Riswanto et al. 2023; Rahayu and Suryanto 2021; Sarı et al. 2017; Suci et al. 2022; Smith and Feng 2018; Tekin 2019; Topçam 2022; Xu 2021; Varita 2017; Yardım 2021; Yıldırım et al. 2019; Zafar et al. 2021; Zhang 2023). These studies have focused on the many different uses of reading circles in different areas, such as the role of fairy tales in reading circles (Kitsanu et al. 2024), academic reading circles (Vartola 2024), the use of reading circles in teaching reading and writing (Yang 2023), the effect on speaking skills (Kaowiwattanakul 2020; King 2001; Matmool and Kaowiwattanakul 2023), the effect of quality conversation in reading circles on thinking skills (Kast 2024), the effect on speaking and pronunciation in foreign language teaching (Liu 2022; Su et al. 2019; Sutrisno et al. 2019; Yakut 2020), the effect on identity development (Le 2021), the effect in the field of nursing (Begoray and Banister 2008), the effect on developing social skills of engineers (Pacheco and Cervantes 2022) and the effect on socio-emotional development (Venegas 2019). The effect of this method in different areas can be evaluated because reading skills are one of the basic tools for acquiring knowledge in every field, as well as comprising multiple other skills that include different language skills and emotional skills.

Although various studies have been conducted in the literature on reading circles, which are one of the high-level reading methods with these qualities, studies conducted with teacher candidates, including the effect of reading circles on the reading process (Çermik et al. 2019; Çetinkaya and Topçam 2019; Doğan et al. 2018, 2019, 2020; Karatay 2017), its effect on internal reading (Rutherford et al. 2009), its effect on critical literacy skills (Baki 2022), its effect on reading culture (Baki 2023), its effect on interaction and meaning-making (Vijayarajoo 2015), its effect on teaching informative text structure (Clark 2021; Diego-Medrano et al. 2016), teacher candidates’ perceptions of reading circles (Aytan 2018), their perceptions of their abilities (Walker and Castillo 2023), social justice (Madhuri et al. 2015) and their perception of social justice (Wexler 2021), their upbringing as representatives of social justice (Davis and Bush 2021), their teaching of the concept of equality (Robertson and Sivia 2025), their social interactions (Calzada 2013), their impact on cultural development (Heineke 2014) and their impact on reflective practices (Ahmed et al. 2024) have also been studied. These studies support the reading circle method as a remarkable reading method that stands out among contemporary educational approaches and offers opportunities for developing reading skills and different social skills in a multifaceted way.

However, the basic key to reading, learning and discovering new things is the sense of curiosity (Yazıcı and Kartal 2020). The importance of the sense of curiosity in education is increasingly emphasized and is accepted as an important incentive element for individuals to actively participate in their own learning processes. Curiosity and discovery, which are key factors that increase individuals’ motivation to learn, also have a role in increasing reading comprehension (Gurning and Siregar 2017). In the research of Kırmızı and Kapıkıran (2019), it was determined that there was a significant relationship at an average level between teacher candidates’ perceptions of curiosity and discovery and their reading habits. Although the reading circle method encourages the sharing of information among readers and provides the opportunity to discover different perspectives and develop original thoughts, it can be said that no research has been found in the literature on the effect of this method, which is woven with various reading strategies, on curiosity and discovery. In addition, the reading circle method encourages readers to approach the text from different perspectives, to reconstruct the text using critical and creative thinking skills and to create new meanings with this learning opportunity that is offered in groups at certain intervals (Daniels 2002). According to Nardelli (2013), the reaction and integration phases of the reading process, which consists of four areas—perception, comprehension, reaction and integration—fall within the scope of creative reading skills. In reading circles, the environment provided for readers to discover different perspectives and create original thoughts is among the elements that support the creativity of readers. Creative reading skills, which are based on creativity and the ability to read between the lines, aim to look for what is different or new in the text and to notice what is not seen in the text, thus ensuring that the development of the sense of curiosity is constantly kept alive during the reading process and feeds the reading process (Al Qahtani 2016; Small and Arnone 2011; Uysal and Coşkun 2023). This is because the active use of creative reading skills in the reading process serves to integrate the ideas and experiences in the texts, to discover hidden meanings and implicit relationships with symbols and signs in the texts, to develop new ideas and to integrate innovations with life (Hızır 2014). The reading process, which can also be called a discovery experience (Zhang and Sun 2011), has been described in two studies on the effects of reading circles on creative writing skills and writing motivations (Helmy 2020) and creative reading skills (Mohamed 2023) in relation to creativity in reading circles, which focus on the reconstruction and creation of the text. It can be said that studies on creativity are limited in the studies conducted on reading circles.

In addition to language skills, another element that supports creativity in the reading process is visuals (Stokes 2005). Today, the cultural language that visuals create for themselves increasingly surrounds every aspect of life, so they come to the forefront as one of the skills that individuals must have (Hattwig et al. 2012; Parsa 2007). In addition, visuals, which are one of the increasingly important elements that support creativity and the reading process today, are important elements that contribute to the concretization of information in texts and the expansion of meaning, as well as the development of aesthetic understanding in readers (Sarıkaya 2017). The skill pattern that enables the active use of visual skills is defined as visual literacy (Elkins 2003; Siegel 2006). The role of the painter (artist), which is among the main roles in reading circles, also focuses on the development of these skills in the reading process. However, when the studies on reading circles are examined, it can be said that no research has been conducted on the effect of visual literacy competencies. In addition, when studies on teacher candidates are examined, it is stated that they do not have the necessary knowledge regarding visual literacy competencies and that the professional development of teacher candidates should be contributed to with methods and techniques that will enable them to develop these skills (Ateş et al. 2020; Çelik and Çelik 2014; Şakiroğlu 2021).

When the existing studies on reading circles are evaluated in terms of research methods, it can be said that the use of this method is surrounded by various experimental or qualitative studies on its effect on reading skills and social skills (Baki 2022, 2023; Çermik et al. 2019; Doğan et al. 2018, 2019; Karatay 2017) and that mixed studies conducted with prospective teachers are limited (Sarı et al. 2017; Tosun 2018). Tavşanlı and Ulaş (2023) stated that the reading circle method is defined by teachers as a fun and effective method, that its use is recommended by teachers and that interest in this method has increased especially after 2015. However, the studies so far focus more on reading comprehension skills and are more quantitative, and in addition to these results, it is emphasized that prospective teachers should also be introduced to the reading circle method. In the research, it was stated that there are limited studies on this method with prospective teachers and that a significant amount of research is still needed in this area; it would be of benefit to see how reading circles affect different skills as well as reading skills and the general framework (Tavşanlı and Ulaş 2023). In the qualitative research conducted by Çetinkaya and Topçam (2019) with eight prospective teachers, the effect of reading circles on enriching the views of prospective teachers about the teaching profession was investigated, and as a result of the research, the participants stated that this method changed their views about the teaching profession positively. The use of the reading circle method in developing the reading skills of teachers and future prospective teachers is emphasized in numerous studies (Allan et al. 2005; Çetinkaya and Topçam 2019; Daniels 2002; Schoonmaker 2014). Based on the view that the reading circle is an important method for the professional development of teachers (Çermik et al. 2019; Çetinkaya and Topçam 2019; Doğan et al. 2019), it can be said that there is a need to teach this method to both teachers and prospective teachers and to examine the effect of this method on their development.

It is anticipated that the examination of the reading circle method’s effect on Turkish teacher candidates’ curiosity and discovery, creative reading skills and visual literacy competencies will make significant contributions to the literature. It is believed that this study will provide teacher candidates with the opportunity to learn a new reading method during their pre-service education and that examining the effects of this method will make significant contributions to their personal development and their use of the method in their professional lives. Within the framework of these objectives, the following problems were sought in the current study:

1. Does the reading circle method create a significant difference between the pre-test and post-test scores of Turkish teacher candidates in creative reading skills?
2. Does the reading circle method create a significant difference between the pre-test and post-test scores of Turkish teacher candidates in curiosity and discovery perceptions?
3. Does the reading circle method create a significant difference between the pre-test and post-test scores of Turkish teacher candidates in visual literacy competencies?
4. What are the views of Turkish teacher candidates regarding the effects of the reading circle method on creative reading skills, curiosity and discovery perceptions and visual literacy competencies?

## 2. Materials and Methods

### 2.1. Research Model

This study was carried out using the mixed-methods approach, which integrates qualitative and quantitative methodologies (Creswell and Clark 2011; Creswell 2017). By enabling the collection and analysis of both types of data, this approach enhances the depth and breadth of the research process. The use of multiple perspectives provided by both paradigms allows for a more nuanced understanding of the data. It can be argued that this method plays an effective role in better understanding research problems (Creswell and Clark 2017). In this study, an exploratory sequential design—a type of mixed-methods design—was employed. This design seeks to combine the strengths and minimize the limitations of both qualitative and quantitative approaches to reduce potential errors in the findings. In this design, quantitative data are collected first, followed by the collection of qualitative data, which are then used to provide a more detailed explanation of the quantitative results (Creswell 2017). In the present study, an experimental intervention was initially implemented, and data related to this intervention were collected. Subsequently, qualitative data were gathered through semi-structured interviews conducted with participants in the experimental group. The findings obtained from the quantitative data were supported by the qualitative findings, and the results of the experimental intervention were evaluated accordingly.

In the quantitative phase of this study, which examined the effects of the reading circle method on pre-service Turkish language teachers’ creative reading skills, perceptions of curiosity and exploration and visual literacy competencies, a pre-test–post-test quasi-experimental design without a control group was employed. The implementation of the reading circle method constituted the experimental intervention, while the participants’ creative reading skills, perceptions of curiosity and exploration and visual literacy competencies, which the intervention aimed to influence, represented the targeted outcomes of this study.

In this study, a control group was not used to comparatively measure the effects of the experimental intervention. The pre-test–post-test quasi-experimental design without a control group involves administering pre-tests and post-tests to the same group before and after the intervention to examine its effects (Büyüköztürk 2007; Sönmez and Alacapınar 2013). While this may limit the generalizability of the findings, dividing participants into different groups was not considered pedagogically or ethically appropriate within the context of this study. To maintain the integrity of the classroom environment, avoid introducing artificial conditions and ensure equal access and opportunity for all students, the same intervention was applied to the entire group. Therefore, a single-group design was preferred (Cohen et al. 2018; Fraenkel et al. 2012).

The single-group pre-test–post-test design allows for the direct observation of differences before and after the experimental intervention (the reading circle method) and can serve as an effective starting point, particularly when implementing a new method or strategy (Creswell 2012). In this study, a control group was not deemed necessary because the effects of the reading circle method on creative reading, visual reading, curiosity and exploration skills were being examined for the first time. Moreover, to enhance the reliability of this study and address the limitation of not including a control group, quantitative data were supported by qualitative data sources—including student posters, semi-structured interviews and expert validation. The findings were interpreted by triangulating these multiple data sources. Thus, the methodological limitations of this study were addressed by supporting the findings with multiple data sources (Yıldırım and Şimşek 2022). In summary, due to ethical considerations and the aim of collecting realistic data in a natural setting, a control group was not included. Instead, pre-test and post-test data were supported by qualitative data and comparisons with previous research to enhance the reliability of the findings (Cohen et al. 2018; Creswell 2012; Fraenkel et al. 2012). For these reasons, the experimental design adopted in this study is presented in Table 1.

In the qualitative dimension of the research, a case study design was used, which allows for detailed and in-depth information to be collected and the situation to be defined in the finest detail. In this design, the cause–effect relationships between variables allow for a detailed and in-depth reflection of the situation by taking a section from the current situation without generalizing (Creswell 2017). In the qualitative dimension of this research, semi-structured interviews were conducted to collect qualitative data, revealing the views of the participants in the experimental group on the effects of the reading circle method on curiosity and discovery, creative reading and visual literacy competencies.

### 2.2. Study Group

In the quantitative phase of this study, participants were selected using a simple random sampling method. The sample consisted of 48 pre-service teachers enrolled in the Department of Turkish Language Education at a university located in the northern region of Türkiye.

There are several reasons for conducting this research specifically within the context of Türkiye. First and foremost, the current Turkish Language Curriculum (MEB 2024) issued by the Ministry of National Education not only emphasizes comprehension skills but also aims to develop students’ creative thinking, visual reading and presentation, multidimensional reading and exploratory learning attitudes. In this context, there is a growing need for effective instructional methods that support the development of these skills (MEB 2024). In this context, examining the effectiveness of student-centered and creative methods such as the reading circle within the specific context of Türkiye is important for assessing the alignment between instructional practices and curriculum goals.

Moreover, previous research has pointed to various shortcomings in the development of reading skills based on visual literacy and creative thinking in Türkiye (Kırmızı 2012) while also emphasizing the limited implementation of practices grounded in creative reading and alternative approaches (Göçer and Garip 2021). Therefore, it is essential to investigate how the reading circle—an approach that is student-centered, collaborative and inquiry-based—impacts the Turkish educational context.

Furthermore, the reading circle method is of Western origin and has primarily been developed and implemented in English-speaking countries. However, since educational methods and strategies cannot be developed independently of their cultural and pedagogical contexts (Paris 2012), factors such as students’ sense of curiosity, visual literacy skills and creative reading abilities may vary in an education system like Türkiye’s, which reflects diverse linguistic and cultural characteristics. In addition, the limited implementation of this method in Türkiye and the need to evaluate the potential impact of cultural differences on its effectiveness have made it necessary to conduct this research in a local context (Paris 2012). Evaluating the functionality and effectiveness of this method is expected to make a unique contribution to the Turkish education system, both academically and in terms of practical application.

Additionally, this research holds a pioneering position in the literature, as it is the first study to holistically evaluate the effects of the reading circle method on creative reading, visual literacy and curiosity-driven exploration skills within the context of Türkiye. In this regard, this study was conducted with pre-service Turkish language teachers, as they are responsible for teaching language skills and there is a need to ensure their high-quality preparation by examining the impact of this method on them. The characteristics of the participants involved in this study are presented in Table 2.

The sample qualitative phase of this research was determined using the purposive sampling method, which is one of the easily accessible case sampling methods. With this sampling method, 43 teacher candidates who participated in the experimental process were determined on a voluntary basis and constituted the qualitative study group. Of the participants in the study group of the qualitative research section, 34 were female and 9 were male. Thus, the effects of the reading circle method on creative reading skills, curiosity and discovery perceptions and visual literacy competencies were examined both qualitatively and quantitatively in the same group.

### 2.3. Data Collection Tools

In collecting the research data, the Creative Reading Skills Scale for Prospective Teachers, Curiosity and Exploration Scale-II (CES), Visual Literacy Competencies Scale and semi-structured interview form were used.

#### 2.3.1. Creative Reading Skills Scale for Prospective Teachers

This measurement tool was developed by Uysal and Coşkun (2023). Both exploratory and confirmatory factor analyses were conducted for the factor structure of this measurement tool, which aims to measure the creative reading skills of prospective teachers. As a result of the analyses, a structure consisting of 6 factors and 33 items, namely, “restructuring the text”, “focusing on the values in the text”, “establishing connections with the characters in the text”, “determining the purposes of creating the text” and “interpreting the text and making predictions about the text”, was reached. The Cronbach Alpha reliability coefficient of the measurement tool was 0.93 for the entire scale; for the subdimensions, the internal consistency coefficients were found to be 0.94 for the “restructuring the text” factor; 0.87 for the “focusing on values in the text” factor; 0.77 for the “establishing connections with characters in the text” factor; 0.78 for the “determining the purposes of the text” factor; 0.73 for the “interpreting the text” factor; and 0.74 for the “making predictions about the text” factor. When the findings obtained as a result of the CFA conducted on the measurement tool were evaluated, the χ^2^/sd ratio was found to be 3.21 (χ^2^/sd = 1533.23/477), RMSEA = 0.07; RMR = 0.08; SRMR = 0.06; CFI = 0.96; IFI = 0.96; and NNFI = 0.96. All these show that the factor loading values of all items as well as the fit values are statistically significant. Thus, the six-factor structure of the measurement tool determined by EFA was confirmed by CFA, and it was determined that it was a valid and reliable measurement tool that could measure the creative reading skills of prospective teachers.

For this study, the Cronbach’s Alpha internal consistency coefficient was calculated to determine the reliability of the entire Creative Reading Skills Scale and its subdimensions. As a result of the reliability analysis conducted on the measurement tool, it was determined that the Cronbach’s Alpha reliability coefficient was 0.80 for the entire scale; the internal consistency coefficients for the subdimensions were 0.79 for the “restructuring the text” factor; 0.82 for the “focusing on values in the text” factor; 0.82 for the “establishing connections with characters in the text” factor; 0.82 for the “determining the purposes of creating the text” factor; 0.82 for the “interpreting the text” factor; and 0.82 for the “making predictions about the text” factor. It can be said that the results of the reliability analysis conducted on the scale were at a satisfactory level.

#### 2.3.2. Curiosity and Exploration Scale-II (CSU)

This measurement tool was developed by Kashdan et al. (2009) and consists of 10 items. Both exploration and confirmatory factor analyses were conducted for the factor structure of the scale. The scale has a two-factor structure. The flexibility subscale (6 items) reflects the motivation to seek knowledge and new experiences; the acceptance of uncertainty subscale (4 items) reflects the desire to explore the uncertain and unpredictable. Indices ranging from good to excellent were obtained regarding the confirmatory factor structure of the scale. The relationship between the two subdimensions of the scale was found to be 0.85. Cronbach’s Alpha coefficients of the scale were calculated to be between 0.75 and 0.86. the Curiosity and Exploration Scale-II (CSU) was translated to Turkish by Acun et al. (2013). As a result of the confirmatory factor analysis of the scale—RMSEA = 0.05; SRMR = 0.04—it was determined that there were good and excellent fit values with CFI = 0.98 and NNFI = 0.97. Cronbach’s Alpha reliability coefficient of the scale was calculated as 0.83 for this study. It can be said that the results of the reliability analysis conducted on the scale were at a good level.

For this study, the Cronbach’s Alpha internal consistency coefficient was calculated to determine the reliability of the Curiosity and Exploration Scale and its subdimensions. As a result of the reliability analysis conducted on the measurement tool, the Cronbach’s Alpha reliability coefficient was calculated as 0.87 for the entire scale; 0.88 for the “flexibility” subdimension; and 0.87 for the “uncertainty” subdimension. It can be said that the results of the reliability analysis conducted on the scale are at a good level.

#### 2.3.3. Visual Literacy Competencies Scale

This measurement tool was developed by Kiper et al. (2012) and named the “Visual Literacy Competencies Scale”. The measurement tool, consisting of 29 items in a 5-point Likert format, consists of six subfactor structures: “being able to give importance to visuality using Office software”, “being able to identify printed visual materials”, “being able to interpret visuals”, “being able to distinguish visual messages encountered in daily life”, “being able to produce visuals using tools” and “being able to perceive messages in visuals”. The Cronbach’s Alpha internal consistency coefficients of these subdimensions were calculated as 0.89, 0.83, 0.86, 0.78, 0.77 and 0.68, respectively, and 0.94 for the entire scale.

For this study, the Cronbach’s Alpha internal consistency coefficient was calculated to determine the reliability of the Visual Literacy Competencies Scale and its subdimensions. As a result of the reliability analysis conducted on the measurement tool, the Cronbach’s Alpha reliability coefficient was calculated as 0.85 for the entire scale; 0.83 for the “ability to give importance to visuality using Office software” subdimension; 0.84 for the “ability to identify printed visual materials” subdimension; 0.84 for the “ability to interpret visuals” subdimension; 0.84 for the “ability to distinguish visual messages encountered in daily life” subdimension; 0.84 for the “ability to produce visuals using tools” subdimension; and 0.84 for the “ability to perceive messages in visuals” subdimension. It can be said that the results of the reliability analysis conducted on the scale are at a satisfactory level.

#### 2.3.4. Semi-Structured Interview Form

In this study, a semi-structured interview form was used to examine the views of Turkish teacher candidates regarding the contributions of the reading circle method’s in-depth reading strategies to the reading process, individual and life skills. In order to prepare the interview form, first of all, the relevant literature was reviewed, and questions were prepared by the researcher. The first section of the form included 3 questions, these questions were presented for the opinions of 2 different field experts and the necessary linguistic adjustments were made depending on the feedback. Then, this form was applied to 5 teacher candidates studying Turkish language teaching. After this pilot study, it was checked whether there was anything unclear in the form. After this application, the questions in the final version of the form are as follows:

1. What are the contributions of the reading circle method to the development of your curiosity and discovery perceptions?
2. What are the contributions of the reading circle method to the development of your creative reading skills?
3. What are the contributions of the reading circle method to the development of your visual literacy skills?

### 2.4. Data Collection

In this study, the nested design, which is one of the mixed research data collection designs, was used. In this design, quantitative and qualitative data can be collected sequentially, simultaneously or both together (Creswell and Clark 2017). This design was preferred because quantitative data were collected first and then qualitative data in this study.

**Figure 1:**
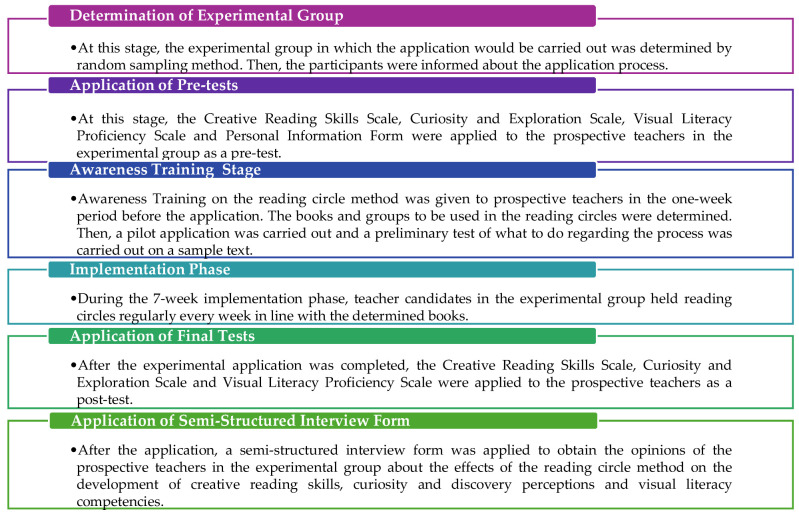
Experimental process.

The collection of qualitative and quantitative data in this study was carried out in two stages. The experimental process lasted 7 weeks, the application of pre-tests and post-tests 2 weeks, Awareness Training 1 week and the semi-structured interviews that constitute the qualitative section 1 week, totaling 11 weeks. To examine the effects of the experimental intervention conducted with the reading circle method, the status of the participants was determined with the scales used as pre-tests and post-tests (Creative Reading Skills Scale, Curiosity and Exploration Scale, Visual Literacy Proficiency Scale) and quantitative data were obtained. To obtain the qualitative data for this study, semi-structured interviews were conducted with the participants in the experimental group. The steps of the experimental process are shown in Figure 1.

**Figure 2:**
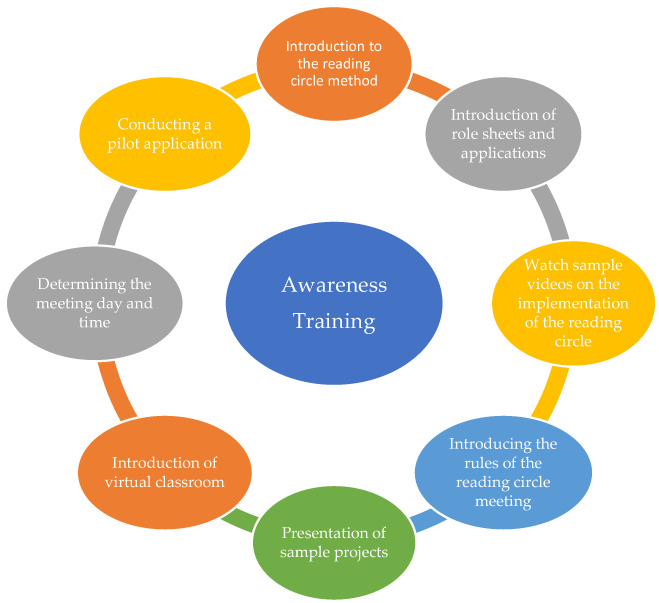
Awareness Training content.

Before the application, the experimental-group students were informed about the research. Then, pre-tests (Creative Reading Skills Scale, Curiosity and Exploration Scale, Visual Literacy Proficiency Scale) were applied. The following week, participants who were to be subjected to the experimental intervention for the applicability of the reading circle method were given Awareness Training on the application of the method. The content of this training is presented in Figure 2.

Awareness Training consisted of a total of 8 h, and a 1-week period was allocated to this training. Introduction to the reading circle method, role sheets and their features, sample application videos related to the reading circle method, rules of reading circle meetings, decision on the projects to be completed each week, creation and introduction of the virtual classroom where the applications were to be followed, introduction of the books to be read, determination of meeting days and hours and formation of groups were provided. In this training, the features of the reading circle method and role sheets were introduced to the prospective teachers; these role sheets were created by the researcher by examining the studies in the literature (Avcı et al. 2010; Baki 2022; Doğan et al. 2018). The prospective teachers in the groups came to the meeting with the notes they took on the worksheets according to their changing roles every week, and after the completion of the relevant meetings, these worksheets were uploaded to a virtual classroom, which was created to make this process easier and to share the principles of the reading circle method, the content of Awareness Education, role sheets and the created applications; the applications were uploaded to the virtual classroom weekly. Announcements were made through this classroom so that prospective teachers could obtain the necessary information about both the method and the role sheets they would use.

In the application of the reading circle method, the selection of books can be left to the students to encourage them to love reading and develop reading habits (Moeller and Moeller 2007). For this reason, the opinions of the prospective teachers participating in the application were taken regarding the books they wanted to read. Then, a list was created regarding the books they wanted to read, and the selection process was carried out with the entire application group. The list of books that were determined as common at the end of this interview is presented in Table 3.

After the selection of books, reading circle groups were formed. In the formation of these groups, a total of 7 groups were formed on a voluntary basis, with a minimum of 6 people per group. Separate interviews were held with each of these groups during the Awareness Training week, and the distribution of roles in the groups was ensured. A name was determined for each group, and introductions were made within the group. The reading circle groups, and their names are presented in Table 4.

After the groups were determined, the weekly meeting day and time were determined and the “Observer”, whose role was to conduct and supervise the meeting, supervised the group activities every week, recorded them on the relevant form and ensured that the role sheets and projects of each group were uploaded to the virtual classroom. In this method, at the end of each application, a project study is carried out that includes a general evaluation of the book through activities such as drama, pantomime, painting, posters, banners, book introduction videos, etc. This was performed through reading circles. After each reading circle, each teacher candidate individually designed posters promoting the book as a project, and these posters formed portfolios for the groups. Sample images of the posters created by the experimental-group teacher candidates regarding the books during the application process are presented below.

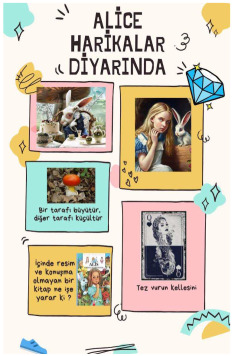

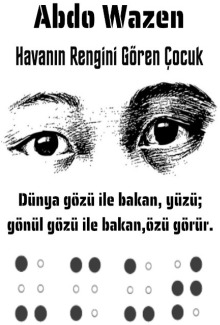

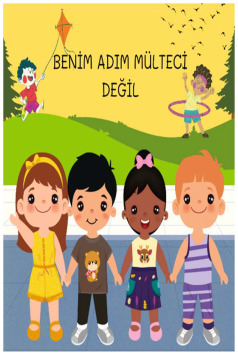
Alice in Wonderland, F_6.The Boy Who Saw the Color of the Weather, M_2.My Name is Not a Refugee, F_18.
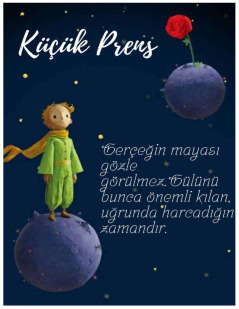

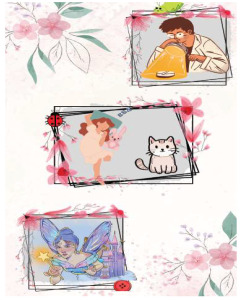

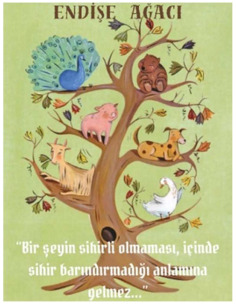
The Little Prince, M_9.Alice in Wonderland, F_20.The Worry Tree, F_26.
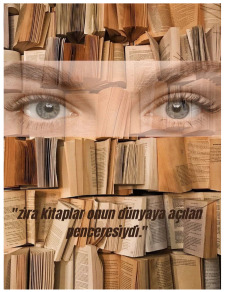

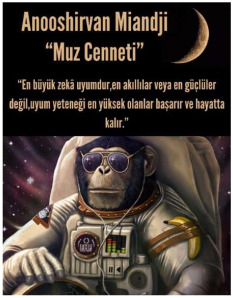

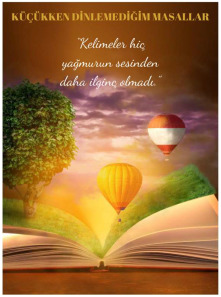
The Boy Who Saw the Color of the Weather, F_29.Banana Paradise, F_17.Fairy Tales I Never Heard When I Was a Child, F_11.
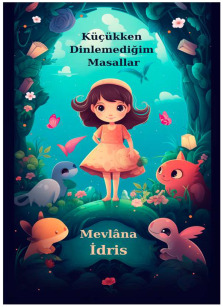

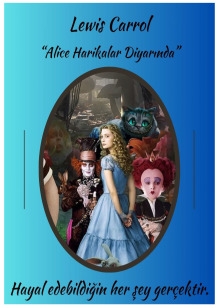

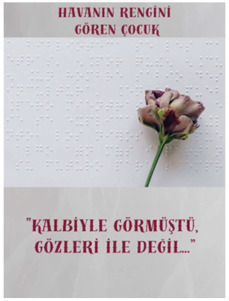
Fairy Tales I Never Heard When I Was a Child, F_30.Alice in Wonderland, F_13.The Boy Who Saw the Color of the Weather, M_8.
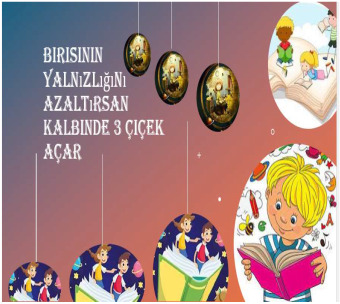

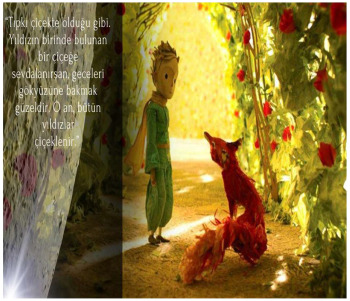
Fairy Tales I Never Heard When I Was a Child, M_3.The Little Prince, F_2.
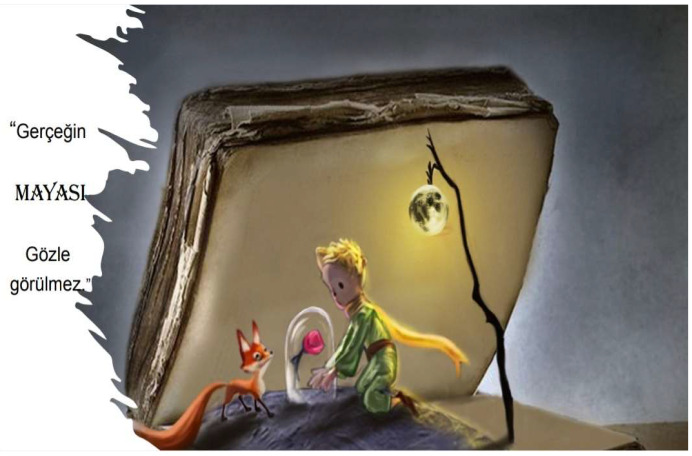
The Little Prince, M_18.

Following the Awareness Training, a pilot application regarding the reading circle was conducted with the teacher candidates using a short text to prevent potential problems during the process. Following this application, the necessary evaluations were made, and the Awareness Training was completed by explaining the points that were not understood in detail.

After the experimental application process, the measurement tools used as a pre-test (Creative Reading Skills Scale, Curiosity and Exploration Scale, Visual Literacy Proficiency Scale) were reapplied to the participants in the experimental group. Semi-structured interviews were conducted the following week, and the interviews lasted an average of 45 min.

### 2.5. Data Analysis

The sequential analysis technique was used in the analysis of the data obtained in this study (Creswell and Clark 2017). The quantitative and qualitative data obtained from this study were analyzed sequentially. In this study, the aim was to collect qualitative data in order to support the results obtained from the data obtained by quantitative means. Thus, bringing together the strengths and weaknesses of two or more research methods reduces the possibility of the researcher making mistakes in this process (Johnson and Christensen 2008). In this technique, quantitative data is first collected and analyzed, then qualitative data is used to explain the quantitative data in more detail (Creswell 2017). In this study, quantitative data were collected and analyzed first, and then semi-structured interviews were conducted with the participants to support the quantitative data. The quantitative data of this study are the data obtained from the pre-test and post-test of the teacher candidates (Creative Reading Skills Scale, Curiosity and Exploration Perception Scale and Visual Literacy Proficiency Scale), and these data were transferred to SPSS (Version 24) after being organized. In the analysis of the data, first, the data-cleaning process was carried out (Tabachnick and Fidel 2007). The assumption of continuity of the data was ensured, and within the framework of the frequency analysis, incorrect coding and data values were checked and arranged from the forms filled in by the participants. Then, the missing data rate in the data set was examined, and values reflecting the mean of the series were assigned for the missing values observed in the data set; next, the scores were converted to standardized z scores in order to determine the extreme values (outliers), and the skewness and kurtosis values of the variables were calculated to examine the univariate normality assumption (Chou and Bentler 1995). Since the data met the normality assumptions, the dependent group t-test analysis was used (Kalaycı 2014).

Content analysis was used to evaluate the data obtained from the semi-structured interviews. This analysis is a systematic, repeatable technique in which some words of a text are summarized with smaller content categories based on certain rules or purposes (Bowen 2009). The basic action in content analysis is to bring together similar data under certain concepts or themes and to organize and interpret them in a way that the reader can understand (Yıldırım and Şimşek 2022). Before this analysis, raw data were categorized and classified as F_1 or F_2 for female participants and M_1 or M_2 for male participants. Each question was considered a theme, the data was analyzed and codes were created. The findings obtained because of this examination were coded according to the themes, and frequency values are presented separately in tables. Direct quotes are included to increase the reliability of the data. To ensure the validity and reliability of this study, the data were examined by two field experts other than the researcher. To increase the reliability of the research, the consistency of the scores as a result of the analysis conducted independently by the researcher and field experts was evaluated with “consensus” or “disagreement”. In the evaluations where the researcher or field experts had conflicting opinions, arrangements were made by reaching a consensus. The reliability of the data analysis in the coding made regarding the scoring was calculated using the formula [Consensus/(Consensus+Disagreement) ×100] (Miles and Huberman 1994). As a result of these coding choices made separately by the field experts at different times, the agreement (reliability) between the opinions of the researcher and the experts was found to be 87% and 82%. This rate shows that a high consistency was achieved between the coders and that this consistency was reliable.

## 3. Findings

In this section, the quantitative and qualitative findings regarding the effects of the reading circle method on the creative reading skills and curiosity and discovery perceptions of Turkish teacher candidates and their visual reading competencies are presented under two separate headings.

### 3.1. Quantitative Findings

At this stage, the quantitative findings regarding the effects of the reading circle method on the creative reading skills and curiosity and discovery perceptions of teacher candidates and their visual reading competencies are presented.

#### 3.1.1. Quantitative Findings Regarding Creative Reading Skills

The t-test results regarding the comparison of the pre-test and post-test scores of teacher candidates from the Creative Reading Skills Scale are given in Table 5.

According to Table 5, it was seen that there was a significant difference between the pre-test score ($\bar{X}$ = 111.12) and post-test score ($\bar{X}$ = 125.00) averages in favor of the post-test scores in the total scale of creative reading skills of the pre-service teachers in the experimental group (t_(47)_ = −4.63, *p <* 0.05). Based on the calculated effect size, it can be said that reading circles have a moderate effect on creative reading skills (d = 0.65).

It was seen that there was a significant difference between the pre-test score ($\bar{X}$ = 43.50) and post-test score ($\bar{X}$ = 47.25) averages in favor of the post-test scores in the dimension of restructuring the text (t_(47)_ = −2.09, *p <* 0.05). Based on the calculated effect size, it can be said that reading circles have a small effect on the text-structuring dimension (d = 0.32).

It was seen that there was a significant difference between the pre-test score ($\bar{X}$ = 14.04) and post-test score ($\bar{X}$ = 16.16) averages in favor of the post-test scores in the dimension of focusing on the values in the text (t_(47)_ = −3.36, *p <* 0.05). Based on the calculated effect size, it can be said that reading circles have a medium-level effect on the dimension of focusing on the values in the text (d = 0.59).

In the dimension of establishing connections with characters in the text, it was observed that there was a significant difference between the pre-test score ($\bar{X}$ = 15.56) and post-test score ($\bar{X}$ = 16.83) averages in favor of the post-test scores (t_(47)_ = −2.33, *p <* 0.05). Based on the calculated effect size, it can be said that reading circles have a small effect on the dimension of establishing connections with the characters in the text (d = 0.45).

In the dimension of determining the purposes of creating the text, it was observed that there was a significant difference between the pre-test score ($\bar{X}$ = 14.66) and post-test score ($\bar{X}$ = 16.47) averages in favor of the post-test scores (t_(47)_ = −3.55, *p <* 0.05). Based on the calculated effect size, it can be said that reading circles have a medium-level effect on the dimension of determining the purposes of creating the text (d = 0.64).

In the dimension of making predictions about the text, it was observed that there was a significant difference between the pre-test score ($\bar{X}$ = 11.62) and post-test score ($\bar{X}$ = 12.52) averages in favor of the post-test scores (t_(47)_ = −2.35, *p <* 0.05). Based on the calculated effect size, it can be said that reading circles have a small effect on the dimension of text-related prediction (d = 0.36).

It was observed that there was no significant difference between the pre-test score ($\bar{X}$ = 11.62) and post-test score ($\bar{X}$ = 12.18) averages in the text interpretation dimension (t_(47)_ = −1.77, *p* > 0.05).

#### 3.1.2. Quantitative Findings Regarding Curiosity and Exploration Perception

The t-test results comparing the pre-test and post-test scores of prospective teachers from the Curiosity and Exploration Perception Scale are given in Table 6.

According to Table 6, it was seen that there was a significant difference between the mean pre-test score ($\bar{X}$ = 29.40) and post-test score ($\bar{X}$ = 32.35) of the Curiosity and Exploration Perception Scale of the pre-service teachers in the experimental group in favor of the post-test scores (t_(47)_ = −3.26, *p <* 0.05). Based on the calculated effect size, it can be said that reading circles have a small effect on the perception of curiosity and exploration (d = 0.46).

It was seen that there was a significant difference between the mean pre-test score ($\bar{X}$ = 20.50) and post-test score ($\bar{X}$ = 22.31) in the flexibility dimension in favor of the post-test scores (t_(47)_ = −2.66, *p <* 0.05). Based on the calculated effect size, it can be said that reading circles have a small effect on the flexibility dimension (d = 0.42).

It was seen that there was a significant difference between the mean pre-test score ($\bar{X}$ = 12.69) and post-test score ($\bar{X}$ = 13.63) in the acceptance of uncertainty dimension in favor of the post-test scores (t_(47)_ = −1.99, *p <* 0.05). Based on the calculated effect size, it can be said that reading circles have a small effect on the dimension of accepting uncertainty (d = 0.28).

#### 3.1.3. Quantitative Findings Regarding Visual Literacy Competencies

The t-test results comparing the pre-test and post-test scores of teacher candidates from the Visual Literacy Competencies Scale are given in Table 7.

According to Table 7, in the total visual literacy competence of the pre-test ($\bar{X}$ = 105.31) and post-test ($\bar{X}$ = 110.90) mean scores, there was a significant difference in favor of the post-test scores (t _(47)_ = −2.12, *p <* 0.05). Based on the calculated effect size, it can be said that reading circles have a small effect on visual literacy competencies (d = 0.30).

In the dimension of being able to identify printed visual materials, there was a significant difference in favor of the post-test scores between the pre-test ($\bar{X}$ = 13.85) and post-test ($\bar{X}$ = 15.00) mean scores (t _(47)_ = −2.30, *p <* 0.05). Based on the calculated effect size, it can be said that reading circles have a small effect on the ability to identify printed visual materials (d = 0.31).

In the dimension of being able to distinguish visual messages encountered in daily life, there was a significant difference in favor of the post-test scores between the pre-test ($\bar{X}$ = 16.37) and post-test ($\bar{X}$ = 20.39) mean scores (t _(47)_ = −7.56, *p <* 0.05). Based on the calculated effect size, it can be said that reading circles have a large effect on the ability to distinguish visual messages encountered in daily life (d = 1.19).

In the dimension of being able to produce visuals using tools, it was observed that there was a significant difference between the pre-test score ($\bar{X}$ = 13.16) and post-test score ($\bar{X}$ = 18.25) averages in favor of the post-test scores (t _(47)_ = 6.71, *p <* 0.05). Based on the calculated effect size, it can be said that reading circles have a large effect on the ability to produce visuals using tools (d = 1.12).

In the dimension of being able to perceive messages in visuals, it was observed that there was a significant difference between the pre-test score ($\bar{X}$ = 10.00) and post-test score ($\bar{X}$ = 11.02) averages in favor of the post-test scores (t (_47)_ = −0.69, *p <* 0.05). Based on the calculated effect size, it can be said that reading circles have a small effect on the ability to perceive messages in visuals (d = 0.40).

In the dimension of being able to give importance to visuals using Office software, it was observed that there was no significant difference between the pre-test score ($\bar{X}$ = 2.85) and post-test score ($\bar{X}$ = 25.85) averages (t _(47)_ = −1.17, *p* > 0.05).

It was observed that there was no significant difference between the pre-test score ($\bar{X}$ = 19.75) and post-test score ($\bar{X}$ = 20.45) means in the visual interpretation dimension (t _(47)_ = −1.03, *p* > 0.05).

### 3.2. Qualitative Findings

At this stage, the findings regarding the views of the reading circle method on the creative reading skills and curiosity and discovery perceptions of Turkish language teachers and their visual reading competencies are presented.

#### 3.2.1. Qualitative Findings Regarding Creative Reading Skills

The views of the teacher candidates regarding the contributions of the reading circle method to the development of creative reading skills are presented in Table 8.

According to Table 8, the contributions of the reading circle method to the development of creative reading skills of prospective teachers are as follows: recreating the text (f = 34), empathizing with the character in the text (f = 32), creative thinking (f = 23), interpreting the text in depth (f = 24), understanding the text in depth (f = 17), creating new meanings from the text (f = 17), understanding the multi-layered structure of the text (f = 16), establishing a connection between the character in the text and life (f = 14), developing imagination (f = 11), analyzing the text structurally (f = 11), increasing the level of focus on the text (f = 10), realizing the multi-layered structure of values (f = 9), examining the text in depth (f = 8), switching from superficial to in-depth reading (f = 7), examining the text from multiple perspectives (f = 7), turning reading into an enjoyable and meaningful experience (f = 6), analyzing the values in the text (f = 6), realizing the importance of values (f = 6), developing critical thinking skills (f = 6), examining the text critically (f = 5), making predictions about the text (f = 5), developing flexible thinking skills (f = 4), determining the purpose of writing the text (f = 4), multidimensional reading skills (f = 4), active participation in the reading process (f = 3), evaluating the characters in the text from multiple perspectives (f = 3) and respecting different opinions (f = 2).

Some of the participants’ views on the impact of reading circles on the development of creative reading skills are as follows:

*By empathizing, we can better understand the motivations and feelings of the characters. *

(M_1)

*Reading circles allow each reader to interpret the text from their own perspective. Seeing that the same text can be perceived in different ways helped me develop my thinking skills and creative interpretations. *

(M_5)

*Different perspectives within the group allowed me to reconsider my own thoughts on the values of the text. *

(M_9)

*The discussions held in the reading circle allowed me to push the boundaries of the text and imagine the text in different ways. *

(M_8)

*The different interpretations of each participant about the text showed me that the text has many more layers of meaning. *

(F_7)

*Thanks to the reading circle, it contributed to the development of our ability to interpret the text from different perspectives. Thus, it allowed us to recreate the text in an original way using our creativity. *

(F_17)

*I can say that while trying to apply every role I took, my creative reading and creative thinking skills also improved. *

(F_14)

*Reading circle is a powerful tool for not only understanding the text but also adding new meanings to it and seeing it from different perspectives. *

(F_16)

*Looking at the events experienced by the characters through their eyes improved my ability to empathize and allowed me to approach the texts I read with stronger connections. *

(F_19)

#### 3.2.2. Qualitative Findings Regarding Curiosity and Discovery Perception

The views of prospective teachers regarding the contributions of the reading circle method to the development of curiosity and discovery perception are presented in Table 9.

According to Table 9, the contributions of the reading circle method to the development of teacher candidates’ perceptions of curiosity and discovery can be listed as follows: desire to discover new things (f = 48), increasing curiosity for learning (f = 32), desire to ask questions and to question (f = 23), developing research skills (f = 16), thinking in depth (f = 16), discovering the power of different perspectives (f = 12), desire to discover the deep structure of the text (f = 15), desire to question and discover oneself (f = 8), expansion of thoughts (f = 4), pleasure of discovery (f = 4), desire to discover the unknown (f = 4), turning the reading process into discovery (f = 4), desire to discover new meanings of words (f = 3), discovering details (f = 3), questioning existence (f = 2) and discovering the pleasure of understanding and questioning (f = 1).

Some of the participants’ views on the effect of the reading circle method on the development of perceptions of curiosity and discovery are as follows:

*The reading circle provided an ideal environment for individuals to listen to each other’s perspectives and learn new things from these perspectives. *

(M_2)

*Curiosity and discovery are basic characteristics inherent in human nature, related to the desire to understand the world around them and learn more. The reading circle supported these basic characteristics and helped me think more deeply and gain a broad perspective. *

(M_6)

*Since everyone has a different perspective, the discussions held in the reading circle encouraged individuals to question their own thoughts more deeply and ask more questions. *

(M_8)

*Listening to different perspectives allowed me to form new thoughts about events or characters. *

(M_4)

*The discussions held in the circle allowed me to discover the underlying messages of the text in more depth. *

(M_7)

*Thanks to the discussions we had in the group, I had the opportunity to question the subtexts and deeper meanings of the text. This process naturally increased my curiosity and allowed me to examine what I read more carefully. *

(F_6)

*I began to understand the book more deeply with the role of the researcher. *

(F_3)

*The reading circle method helped me question and discover more. Thus, it contributed to me being more interested, inquisitive and curious about learning. *

(F_11)

*The reading circle helped us find our own meanings by exploring the text. *

(F_3)

*I think the literature circle makes books more exciting and fuller of discovery because hearing different perspectives from everyone increases the sense of curiosity. *

(F_4)

*The reading circle method transforms the reading process from being just a process of acquiring information to a process of discovery, interpretation and production. *

(F_22)

#### 3.2.3. Qualitative Findings Regarding Visual Literacy Competencies

The views of prospective teachers regarding the contributions of the reading circle method to the development of visual literacy competencies are presented in Table 10.

According to Table 10, the contributions of the reading circle method to the development of visual literacy competencies of prospective teachers can be listed as follows: visual production skills (f = 42), establishing semantic relationships between visuals and texts (f = 33), analyzing visual messages in daily life (f = 32), analyzing and interpreting visuals in printed texts (f = 28), realizing the importance of visuals in daily life (f = 27), critically examining visuals in social life (f = 26), being consciously visually literate (f = 20), realizing the importance of visuals in printed texts (f = 17), visual perception skills (f = 16), being able to analyze images and symbols in visuals (f = 13), being able to analyze the design features of visuals (f = 7) and visual creativity and aesthetic sensitivity (f = 5).

Some of the participants’ views on the effect of the reading circle method on the development of visual literacy competencies are as follows:

*Analyzing book covers, illustrations or graphics strengthened our visual perception. *

(M_6)

*This method has encouraged me to not only read texts but also to examine the visuals in the text in depth. *

(F_3)

*Understanding how images relate to text has significantly increased my visual literacy. *

(F_7)

*Being able to establish a connection between the content of the visuals and the text helped me correctly define their messages. *

(F_20)

*The reading circle provided me with a more critical perspective not only on books but also on visual content in magazines, advertisements and social media. *

(F_9)

*It allowed me to strengthen my own narrative by using visual tools. *

(F_11)

*My ability to distinguish visual messages I encounter in daily life has increased; for example, I can better understand the meanings in advertisements or posters. *

(F_17)

*I can get to know printed materials better by analyzing the cover designs and images inside the books. *

(F_29)

*It is now much easier for me to perceive visual messages and analyze the visuals I encounter in daily life. *

(F_5)

*The reading circle is a versatile method that supports me not only in understanding written texts but also in developing my visual literacy skills. My ability to recognize visual materials has improved. *

(F_3)

*In the reading circle, I improved my ability to interpret the relationship between visuals and the text. *

(F_7)

*I learned to consider visuals not only as an aesthetic element but also as an important element that produces meaning and needs to be analyzed with the text. *

(F_22)

*I think the reading circle greatly contributes to the development of my visual literacy skills because it helped me make sense of the texts by supporting them not only with words but also with visuals. *

(F_8)

*I can more easily notice the hidden meanings in advertisements, manipulative visuals in the news, or misleading posts on social media. *

(F_4)

## 4. Discussion

This study examined the impact of the reading circle method on pre-service Turkish language teachers’ creative reading skills, perceptions of curiosity and exploration and visual literacy competencies. The findings revealed that the reading circle method significantly improved the participants’ creative reading skills. Similarly, Mohamed’s (2023) study with pre-service teachers also found that reading circles significantly enhanced creative reading abilities. Further analysis of the subdimensions of creative reading skills indicated that reading circles had a notable effect on structuring the text, identifying the purpose of the text and making predictions related to the text. The qualitative findings of this study revealed that the reading circle method contributed most significantly to recreating the text, creative thinking, generating new meanings and the development of imagination. Similarly, previous studies have shown that this method enhances pre-service teachers’ creative thinking skills (Al Qahtani 2016; Çermik et al. 2019) and imaginative abilities (Baki 2023). Since creative reading is considered an outcome of the use of creative thinking skills (Priyatni and Martutik 2020; Yurdakal 2019), it can be argued that this method also fosters a variety of skills that support creative reading. Indeed, the qualitative data indicated that the method also contributes to the development of other skills such as critical thinking and flexible thinking. These findings are consistent with previous research indicating that discussions within reading circles significantly enhance critical thinking skills (Baki 2022; Çermik et al. 2019; Karatay 2017; Matmool and Kaowiwattanakul 2023; Pambianchi 2017).

Meaning-making, the most crucial stage of the reading process, involves the unfolding of meaning from the surface structure (concrete level) to the deep structure (abstract level) (Onan 2015). Creative reading, on the other hand, goes beyond simply connecting the surface and deep structures—it involves discovering new and different meanings within a text and reconstructing the text itself (Litman 2017; Priyatni and Martutik 2020). The qualitative findings of this study revealed that the reading circle method contributed to deep understanding of texts, analyzing the multi-layered structure of meaning and shifting from surface-level to in-depth reading. Previous studies have also shown that this method helps pre-service teachers identify the theme and main idea of a text (Karatay 2017), enrich their vocabulary (Aytan 2018; Çermik et al. 2019) and improve their deep/inferential comprehension (Avcı et al. 2013; Baki 2023; Levy 2019; Mete 2020). Moreover, it can be stated that this method is a multifaceted approach that supports the development of text examination, comprehension, analysis and evaluation skills (Clark 2021; Karatay and Soysal 2021), as well as overall reading comprehension abilities (Goa and Wodai 2022; Liu 2022; Novasyari 2021; Nurhadi 2017; Purifico 2015; Rahman 2022; Riswanto et al. 2023; Varita 2017; Vijayarajoo 2015).

In the creative reading process, the reader’s emotional responses, which emerge from connections established with characters in the text or with their own prior knowledge, are among the factors that can influence the reading process either positively or negatively (Nardelli 2013). In this study, the evaluation of the subdimensions of creative reading skills revealed that reading circles had a significant impact in terms of focusing on values within the text and establishing connections with the characters. Studies have shown that active participation in reading circles enables readers to establish connections between the text and their prior knowledge (Cloonan et al. 2020; Mrak 2021; Whittingham 2013), as well as between the text and their own experiences and lives (Ahmed et al. 2024; Baki 2023; Tosun and Doğan 2020). It can be stated that this outcome is also influenced by the connecting role of the method, which helps readers interact with what they have learned by linking it to their personal lives. Indeed, the qualitative findings of this study indicate that the method contributes positively to the development of empathy with characters, multidimensional evaluation of characters, building connections between characters and real life, analyzing values in the text and recognizing the significance of those values. Studies in the literature have also shown that reading circles contribute positively to pre-service teachers’ respect for diversity (Doğan et al. 2019), the development of their self-efficacy beliefs (Özbay and Kaldırım 2015) and improvements in self-esteem, self-confidence, motivation and empathy (Ahmed et al. 2024; Aytan 2018; Kim 2004; Monaghan 2016). Additionally, reading circles have been found to enhance self-regulation (Baki 2022; Su et al. 2019), the multidimensional analysis of characters (Çetinkaya and Topçam 2019) and the ability to adopt different perspectives (Aytan 2018; Çermik et al. 2019; Çetinkaya and Topçam 2019; Doğan et al. 2019; Tracey and Morrow 2006). It can be stated that reading circles, through their positive contributions to the development of socio-emotional skills (Brown 2015; Venegas 2019), promote the emotional factors essential for creative reading by activating various emotions and modes of thinking (King 2001; Sarı et al. 2017). The internalization of the text and the emotionally supportive environment created for creative reading were found to enhance affective variables of reading, such as reading motivation (Meredith 2015; Smith and Feng 2018) and attitudes toward reading (Qie et al. 2023).

In the qualitative findings obtained from this study, it was determined that half of the pre-service teachers showed improvement in interpreting the text in depth, whereas the quantitative findings revealed no statistically significant difference in the interpretation subdimension of the Creative Reading Scale. This discrepancy may stem from several reasons. One possible explanation is the divergent results produced by the quantitative and qualitative data obtained from this mixed-methods study. While the quantitative dimension of this study produced generalizable results based on specific scales, the qualitative analysis revealed participants’ experiences and perceptions. One probable reason for this discrepancy may be the differences in measurement tools and methods. Since scales are limited to specific items, they may fall short in fully capturing the cognitive and affective experiences that occur during the reading process (Creswell and Clark 2011). Moreover, the items in the scale may have reflected only certain aspects of text-structuring skills, whereas the interviews with participants might have allowed them to explain how they structured the text from a broader perspective. Another possible reason could be the individual differences in perception and expression among the teacher candidates, as they may have preferred to express themselves in a more personal and contextual language. Additionally, it is possible that the development of text-structuring skills occurred indirectly, which may not have been fully captured by the scale. During the group discussions, activities were conducted to identify the main idea and supporting details of the text; however, the measurement tool used may not have assessed these elements explicitly. Nevertheless, the participants may have recognized and expressed this development themselves. In the interviews, the teacher candidates conveyed their metacognitive awareness, which may not have been directly captured by the measurement tool (Patton 2015). Furthermore, reading circles create socially interactive learning environments, which differ from individual reading processes. In this context, the skill of structuring the text may have developed through social interaction; however, the measurement tool used in this study may not have directly captured the contribution of such interaction to individual learning. Additionally, the characteristics of the reading circle method itself may have influenced this outcome. Specifically, structuring a text requires the reader to construct a conceptual framework, establish intertextual connections and create a coherent structure (Kintsch 1998). Although reading circles contribute to individual meaning-making processes, it can be argued that students may require more systematic guidance and structured reading strategies during the process of text construction. Secondly, in reading circles, students often focus on interpreting the text and providing emotion-based responses to what they have read. This is because one of the foundational principles of this method is reader-response theory (Hsu 2004). While this process may support the development of other dimensions of creative reading skills, it may not sufficiently promote the development of text construction skills (McElvain 2010). Since the development of text construction skills requires analytical and cognitive strategies, it can be suggested that incorporating specific reading strategies under teacher guidance into the process may contribute to the enhancement of this particular skill. Another reason may be that the reading process in reading circles is discussion-oriented rather than individual, which may limit the development of text construction skills. In order for reading circles to support text construction, it is recommended to use various tools to identify the structural schema of the text, including the main idea and supporting details, or to incorporate these elements into appropriate role sheets (Duke and Pearson 2017). Based on these findings, it can be stated that reading circles indirectly contribute to the text construction dimension of creative reading skills, and that this contribution needs to be supported through different methods and strategies in order to be made explicit.

When the findings regarding the effect of reading circles on creative reading skills are evaluated, it is observed that this method has a moderate to moderately strong and significant effect on the overall creative reading skills, particularly in focusing on the values embedded in the text and identifying the purposes for which the text was constructed. In other words, reading circles can be considered an effective, noteworthy and applicable method that leads to significant improvement in overall creative reading skills—particularly in focusing on the values embedded in the text (i.e., understanding the emotions, values and messages) and identifying the purposes for which the text was constructed (i.e., making inferences about the author’s intent and the text’s purpose). However, the method appears to have a relatively small effect on restructuring the text, making predictions about the text and establishing connections with the characters. In other words, it can be stated that the pre-service teachers demonstrated improvement in skills such as organizing the text and establishing logical structure, as well as in making predictions related to the text; however, these improvements remained limited to small effect sizes. Regarding the ability to connect with characters in the text, the method yielded a low-to-moderate effect, approaching the medium level. In other words, it can be concluded that reading circles led to a meaningful but partial improvement in emotional interaction skills such as empathizing with characters, though this effect was not particularly strong. In summary, it can be stated that reading circles had a more limited effect in terms of these three skills. Therefore, in order to enhance the development of these specific skills and strengthen the overall effectiveness of the method, it is necessary to restructure the implementation process by incorporating supportive interventions that include different or additional strategies.

According to another finding of this study, reading circles significantly influenced teacher candidates’ overall perceptions of curiosity and exploration, particularly in the subdimension of flexibility and acceptance of uncertainty. Although no previous studies have been found in the relevant literature specifically examining the effect of this method on curiosity and exploration perceptions, the qualitative findings support these results. Data obtained from the interviews indicated that reading circles primarily enhanced teacher candidates’ desire to discover new things, increased their curiosity for learning and improved their skills in questioning and inquiry. Similarly, in the study conducted by Doğan et al. (2019), it was found that this method improved teacher candidates’ inquiry and thinking skills. According to Avcı et al. (2013), chapter discussions within reading circles, the rotation of roles in each session and the process of reaching conclusions through reflection are among the key factors contributing to this outcome. These effects can be interpreted as a result of the “inquirer” role and the impact of group discussions. In this method, three types of questions are used: recall/closed-ended, interpretive/open-ended and personal. It is particularly expected that the reader in the inquirer role will prefer interpretive questions (Moeller and Moeller 2007). In this study, the development of curiosity and exploratory perception may be attributed to the fact that the reader in the inquirer role chose interpretive questions, which aim to foster multidimensional thinking and allow for multiple responses. Indeed, qualitative findings from this study highlight that this method supports the development of research and deep-thinking skills, as well as the discovery of the value of diverse perspectives. These findings are also supported by previous research suggesting that this method, composed of actions that promote research skills (Daniels 2002), enhances willingness to gather information (Baki 2022), contributes to the development of information acquisition skills (Doğan et al. 2021) and improves research skills (Amalia 2025). Doğan et al. (2019) found that this method, by providing opportunities for the use of higher-order thinking skills such as inquiry, contributed to the development of teacher candidates’ thinking skills through gaining and broadening different perspectives. These findings can be interpreted as a result of the theoretical foundations upon which reading circles are based—namely, reader-response theory, schema theory and inquiry-based learning theory (Daniels 2002; Hsu 2004; McElvain 2010).

Although various methods are used to satisfy an individual’s curiosity and desire for exploration, one of the most practical tools for discovering oneself and existence is reading. Among the prominent themes identified in the qualitative findings of this study are teacher candidates’ discovery of the deep structure of the text, uncovering new meanings of words and transforming the reading process into an act of exploration. In other words, it can be said that teacher candidates described reading as both an enjoyable and meaningful experience while also perceiving it as a tool for exploration through this method. Tosun and Doğan (2020) also revealed that reading circles enhance aesthetic reading skills—that is, the ability to derive pleasure from reading. Indeed, several studies have found that this method positively influences reading enjoyment and curiosity (Baki 2023), as well as increases interest in reading (Amalia 2025; Doğan et al. 2021). The significant relationship between teacher candidates’ sense of curiosity and exploration and their attitudes toward reading (Kırmızı and Kapıkıran 2019) also supports this finding. Among the qualitative findings of the present study, it was further noted that the process guided participants toward discovering the unknown, as well as toward self-discovery and an exploration of existence. Similarly, the contributions of reading circles to teacher candidates in terms of uncovering hidden talents (Aytan 2018) and enhancing attention to detail (Pambianchi 2017) are also consistent with these findings. This may be attributed to the fact that reading circles are grounded in a reader-centered approach based on reader-response theory (Daniels 2002). Taken together, these results suggest that reading circles have a positive impact on fostering individuals’ sense of curiosity and discovery and that they serve to enhance teacher candidates’ intrinsic motivation to explore and inquire through reading.

When the findings regarding the impact of reading circles on the perception of curiosity and exploration are evaluated, it can be stated that the method has a limited yet meaningful effect approaching a moderate level on the overall perception of curiosity and exploration. In other words, this method can be considered to have a significant but limited impact on teacher candidates’ willingness to inquire, ask questions and seek new knowledge. It can be suggested that this method has a small but significant effect on flexibility-related skills such as being open to different perspectives and restructuring one’s thinking. In terms of the dimension of tolerating uncertainty—namely, coping with ambiguity and accepting multiple meanings—it appears to provide a more limited contribution, and this effect can be considered to remain at a minimal level. Based on these findings, it can be concluded that reading circles can be used as a supportive tool that significantly enhances pre-service teachers’ sense of curiosity and exploration. However, in order to increase this effect, it is necessary to supplement the method with different strategies and practices.

Another finding obtained from the research indicates that the reading circle method produced a statistically significant difference in the overall visual literacy competencies of pre-service teachers, as well as in the subdimensions of defining printed visuals, distinguishing visual messages in daily life, producing visuals using tools and perceiving messages in visuals. According to the qualitative findings obtained from the research, it can be stated that this method positively contributes to the development of conscious visual literacy among pre-service teachers by enhancing their abilities to produce visuals, make sense of visuals, analyze and interpret visual messages and recognize the importance of visuals. When the qualitative and quantitative findings are evaluated together, it can be concluded that the contributions provided by reading circles enabled pre-service teachers to evaluate visuals as texts, because like texts, visuals possess a multi-layered structure, and reaching the true meaning in visuals requires a conscious decoding of these layers (Parsa 2007). In this method, the acquisition of such gains may also be influenced by the role of the illustrator (artist), who is responsible for conveying what has been understood from the text through images, graphics, diagrams or charts (Bernadowski and Morgano 2011). Similarly, Doğan et al. (2018) state that the illustrator role provides an opportunity to use visuals as an effective strategy for making sense of the texts being read, which in turn has a positive impact on the development of reading skills. Brabham and Villaume (2000) also found that the illustrator role positively influences participants’ ability to create visual imagery, comprehend textual meanings and construct personal interpretations. Furthermore, it can be stated that teacher candidates’ use of the visual strategy of creating images with visual tools and designing promotional posters for each book in the reading circles contributed significantly to the effectiveness of the implementation. Indeed, Doğan et al. (2019) found that discussions about visuals in books and the use of visuals enhanced teacher candidates’ concretization skills and imagination, thereby creating a more effective reading environment and contributing to more permanent learning. In fact, proficient readers often use various strategies to comprehend texts, such as visualizing the content and making connections between the text and real-life experiences (Brabham and Villaume 2000; Brownlie 2005). Similarly, studies conducted with teacher candidates have shown that reading circles contribute to various visual literacy gains, such as enhancing visual thinking, interpreting and analyzing visuals and structuring visual information (Baki 2023), as well as improving photography skills (Çermik et al. 2019). The qualitative findings of this study also revealed that this method fosters the ability to interpret visual images and symbols and supports the development of visual creativity and aesthetic appreciation. These findings can also be interpreted as an indication of an increased awareness among participants regarding the figurative and symbolic power of visuals. Previous studies have shown that this method enhances teacher candidates’ imagery abilities (Baki 2023) and aesthetic appreciation (Aytan 2018). Based on these findings, it can be stated that reading circles contribute not only to the cognitive dimension of visual literacy skills but also to the development of their affective dimension. Bernadowski and Morgano (2011) also emphasize that the rotation of roles in each session enables readers to experience every role, thereby fostering the development of all reading-related skills, including verbal, written and visual literacy.

According to the quantitative findings of this study, this method did not yield a statistically significant difference in the dimensions of “valuing visuals through Office software” and “interpreting visuals” within the Visual Literacy Proficiency Scale. However, the qualitative findings revealed that more than half of the prospective teachers expressed that the method contributed to their ability to analyze and interpret visuals in printed texts. In Baki’s (2023) study, it was found that this method contributed most significantly to the development of prospective teachers’ ability to interpret and make meaning of visuals among the various visual reading skills. In reading circles, not only drawing but also digital designs can be used to visually convey the meaning derived from the texts (Bernadowski and Morgano 2011). This may be due to the fact that, in the current study, prospective teachers preferred to use Web 2.0 tools rather than Office software while designing posters. Moreover, this may also be attributed to the fact that, in addition to Web 2.0 tools, artificial intelligence tools are more suitable than Office software for designing more effective visuals. Additionally, this situation may have resulted from the research methodology itself. As a matter of fact, in mixed-method research, it is possible for quantitative and qualitative findings to yield contradictory or non-overlapping results; however, such discrepancies offer an opportunity for a deeper understanding of the findings (Teddlie and Tashakkori 2009). This situation may also stem from the limitations of the measurement instrument, which might have been insufficient in capturing the participants’ experiences in depth. In this context, the visual interpretation dimension being assessed consists of abstract and multi-layered skills that are subjective and vary across individuals; therefore, the scale items may have been inadequate in reflecting these aspects (Mertens 2019). Another possible reason is that participants may not have been aware of these skills prior to the implementation, and as a result, this might not have been reflected in the scale responses. However, during the interviews, when they had the opportunity to reflect on these concepts and share their experiences, these skills may have become more visible. Additionally, considering that visual literacy is an interpretive and creative skill, the use of open-ended questions and reflective expressions might have been more functional for its assessment compared to the existing scale. When the current inconsistency is considered in terms of reading circles, it may be that the socially interactive environment provided by this method fostered greater development in the interpretation and comprehension of visuals; however, the scale used might not have been sufficiently sensitive to capture this change.

When the results regarding the effects of reading circles on visual literacy competencies are evaluated, it can be stated that this method had a large effect and led to a remarkably high level of improvement particularly in distinguishing visual messages and in visual production skills. In other words, it can be stated that this method is effective and applicable in terms of providing significant improvement in the skills of analyzing and interpreting visual messages as well as producing visuals using digital tools. On the other hand, the method appears to have a low but meaningful effect on recognizing printed visuals, perceiving the messages in visuals and overall visual literacy competencies. Furthermore, it was found that reading circles did not have a significant impact on presenting visuals using Office software or on visual interpretation. Based on this, it can be concluded that while the method influences certain dimensions of visual literacy competencies, its impact on other dimensions is insignificant. Overall, the method exerts a low-level yet meaningful and limited effect on general visual literacy competencies. To enhance this limited effect and contribute more significantly to the development of these competencies, it is recommended that the method be supported with various visual strategies and digital tools.

## 5. Limitations

The findings of the present study should be interpreted considering certain limitations. This research is limited to the measurement tools used to assess the impact of the reading circles method on pre-service teachers’ creative reading skills, perceptions of curiosity and exploration and visual literacy competencies, as well as to the semi-structured interviews conducted on these topics. Future studies may examine the effects of this model on these variables by conducting research at different educational levels (e.g., primary, secondary, high school). As this study employed a mixed-methods design, it included both qualitative and quantitative data. Through future action research, the effects of the reading circle method on these variables and ways to enhance these effects can be investigated. Additionally, by diversifying the measurement tools used, the impact of various roles within the reading circles can be explored.

## 6. Conclusions and Recommendations

In this study, which examined the effects of the reading circle method on pre-service teachers’ creative reading skills, perceptions of curiosity and exploration and visual literacy competencies, it was found that the implemented reading method had a statistically significant impact on all variables as a result of the experimental application. Based on these findings, it can be concluded that the reading circle method is an effective approach for enhancing pre-service teachers’ creative reading skills, fostering their curiosity and exploratory perceptions and improving their visual literacy competencies. As a result of the research, it can be stated that the reading circle method significantly improved teacher candidates’ overall perception of curiosity and exploration, as well as its subdimensions. Regarding creative reading skills, the method had a significant effect on all subdimensions and the overall score of the measurement tool except for the dimension of interpreting the text. However, in the interviews conducted, about half of the teacher candidates reported an improvement in their ability to interpret texts in depth. Based on these findings, it can be suggested that the reading circle method has an indirect effect on supporting text construction skills. Therefore, incorporating strategies into the role cards used during the implementation of the method—strategies that promote the development of text construction processes—may further enhance this skill. Moreover, the contribution of reading circles to the skill of constructing meaning from a text may not always be clearly measurable through standard assessment tools. Therefore, to comprehensively evaluate the effect of this method on text construction, it is recommended to use both quantitative and qualitative research methods in combination. Additionally, to support the development of text construction skills, it may be beneficial to include more activities within reading circles that allow for schema building, summarizing and establishing intratextual connections under the guidance of the teacher.

In the quantitative findings of this study, reading circles did not produce a statistically significant difference in the subdimensions of the visual literacy scale related to the ability to emphasize visual elements and interpret visuals using Office software. However, the qualitative findings revealed that the method made notable contributions in terms of analyzing and interpreting visuals in printed texts, interpreting visual messages in daily life and critically examining visuals encountered in social contexts. This situation can be interpreted as an indirect contribution of reading circles to visual interpretation skills. It can be argued that multidimensional and development-oriented skills such as visual literacy should not be evaluated solely through quantitative data but rather supported by qualitative data that reflect participants’ experiences, perceptions and thoughts, allowing for a more detailed and in-depth understanding.

In conclusion, the reading circle method can be considered a multidimensional approach that supports the development of pre-service teachers; therefore, future studies may explore this method more comprehensively by incorporating the cognitive and affective skills embedded in the roles assigned during its implementation. Studies focusing on the limitations of the role sheets and their characteristics used in this research may also contribute to the further development of the method. Given its features, the reading circle can be described as a multifaceted method that not only enhances reading skills but also simultaneously supports the development of various other competencies in teacher education. It holds significant potential for improving the quality of teacher training by fostering multiple skills in an integrated and meaningful way.

## Figures and Tables

**Table 1:** Experimental design used in this study.

Group	Pre-Test	Method	Post-Test
G_d_	O_1.1_	X	O_2.1_
	1 week	7 weeks	1 week

Note: Gd: experimental group; O_1.1_: pre-tests to be applied to the experimental group; O_1.2_: post-tests to be applied to the experimental group; X: awareness training and implementation of the reading circle method.

**Table 2:** Characteristics of the study group.

Features		Distribution	
		*f*	%
Gender	Female	37	77.08
	Male	11	22.91
Age	18–20	44	91.66
	21–23	4	8.33
Class	2nd grade	48	100

**Table 3:** Books read in the experimental process regarding the reading circle method.

Title of the Book	Author of the Book
*Alice in Wonderland*	Lewis Carroll
*The Little Prince*	Antoine de Saint-Exupéry
*The Boy Who Saw the Color of the Weather*	Abdo Wazen
*The Worry Tree*	Marianne Musgrove
*Banana Paradise*	Anooshirvan Miandji
*Fairy Tales I Never Heard When I Was a Child*	Mevlâna İdris Zengin
*My Name Is Not a Refugee*	Kate Milner

**Table 4:** Names of reading circle groups in this study.

Groups
Group 1: Suwayda
Group 2: Huma
Group 3: Torch
Group 4: Full Moon
Group 5: Magnolia
Group 6: Circle of Dreams
Group 7: Claret Red

**Table 5:** Dependent group t-test results regarding the pre-test and post-test scores of creative reading skills.

Creative Reading Skills	Group	N	$\bar{X}$	SD	df	t	*p*	Cohen’s d
Restructuring the text	Pre-test	48	43.50	12.30	47	−2.09	0.02	0.32
	Post-test	48	47.25	10.92				
Focusing on the values in the text	Pre-test	48	14.04	3.73	47	−3.36	0.00	0.59
	Post-test	48	16.16	3.43				
Establishing a connection with the characters in the text	Pre-test	48	15.66	2.47	47	−2.33	0.02	0.45
	Post-test	48	16.83	2.70				
Determining the purposes of creating the text	Pre-test	48	14.66	2.74	47	−3.55	0.01	0.64
	Post-test	48	16.47	2.85				
Interpreting the text	Pre-test	48	11.62	2.16	47	−1.77	0.08	-
	Post-test	48	12.18	2.30				
Making predictions about the text	Pre-test	48	11.62	2.54	47	−2.35	0.02	0.36
	Post-test	48	12.52	2.37				
Total of the scale	Pre-test	48	111.12	20.55	47	−4.63	0.00	0.65
	Post-test	48	125.00	21.59				

**Table 6:** Dependent group *t*-test results regarding curiosity and exploration perception pre-test and post-test scores.

Curiosity and Exploration Perception	Group	N	$\bar{X}$	SD	df	t	*p*	Cohen’s d
Flexibility	Pre-test	48	20.50	3.95	47	−2.66	0.02	0.42
	Post-test	48	22.31	4.62				
Accepting uncertainty	Pre-test	48	12.69	3.02	47	−1.99	0.00	0.28
	Post-test	48	13.63	3.54				
Total of the scale	Pre-test	48	29.40	5.87	47	−3.26	0.02	0.46
	Post-test	48	32.35	6.85				

**Table 7:** Dependent group t-test results regarding visual literacy competencies pre-test and post-test scores.

Visual Literacy Proficiency	Group	N	$\bar{X}$	SD	df	t	*p*	Cohen’s d
Being able to give importance to visuality using Office software	Pre-test	48	24.85	5.33	47	−1.17	0.24	-
	Post-test	48	25.85	5.91				
Being able to identify printed visual materials	Pre-test	48	13.85	3.45	47	−2.03	0.04	0.31
	Post-test	48	15.00	3.90				
Being able to interpret visuals	Pre-test	48	19.75	3.73	47	−1.03	0.30	-
	Post-test	48	20.45	3.82				
Being able to distinguish visual messages encountered in daily life	Pre-test	48	16.37	2.89	47	−7.56	0.00	1.19
	Post-test	48	20.39	3.77				
Being able to produce visuals using tools	Pre-test	48	13.16	3.54	47	−6.71	0.00	1.12
	Post-test	48	18.25	5.32				
Being able to perceive messages in visuals	Pre-test	48	10.00	2.22	47	−2.69	0.01	0.40
	Post-test	48	11.02	2.80				
Total of the scale	Pre-test	48	105.31	16.59	47	−2.12	0.03	0.30
	Post-test	48	110.90	21.19				

**Table 8:** Contributions of the reading circle method to the development of creative reading skills.

Main Theme	Sub-Themes	f
Creative reading skills	Recreating the text	34
	Empathizing with the character in the text	32
	Creative thinking	23
	Interpreting the text in depth	24
	Understanding the text in depth	17
	Creating new meanings from the text	17
	Understanding the multi-layered structure of the text	16
	Establishing a connection between the character in the text and life	14
	Development of imagination	11
	Analyzing the text structurally	11
	Increasing the level of focus on the text	10
	Realizing the multi-layered structure of values	9
	Analyzing the text in depth	8
	Moving from superficial to in-depth reading	7
	Analyzing the text in multiple ways	7
	Transforming reading into an enjoyable and meaningful experience	6
	Analyzing the values in the text	6
	Realizing the importance of values	6
	Developing critical thinking skills	6
	Critical examination of the text	5
	Making predictions about the text	5
	Developing flexible thinking skills	4
	Determining the purpose of writing the text	4
	Multidimensional reading skills	4
	Active participation in the reading process	3
	Evaluating the characters in the text in multiple ways	3
	Respecting different opinions hearing	2

**Table 9:** Contributions of the reading circle method to the development of curiosity and discovery perception.

Main Theme	Sub-Themes	f
Curiosity and perception of exploration	Desire to discover new things	48
	Increasing curiosity for learning	32
	Desire to ask questions and to question	23
	Developing research skills	16
	Thinking in depth	19
	Desire to discover the deep structure of the text	15
	Discovering the power of different perspectives	12
	Desire to question and discover oneself	8
	Expansion of thoughts	4
	Pleasure of discovery	4
	Desire to discover the unknown	4
	Transforming the reading process into discovery	4
	Desire to discover new meanings of words	3
	Discovering details	3
	Questioning existence	2
	Discovering the pleasure of understanding and questioning	1

**Table 10:** Contributions of the reading circle method to the development of visual literacy competencies.

Main Theme	Sub-Themes	f
Visual literacy competence	Visual production skills	42
	Establishing a semantic relationship between visuals and text	33
	Analyzing visual messages in daily life	32
	Analyzing and interpreting visuals in printed texts	28
	Realizing the importance of visuals in daily life	27
	Critically examining visuals in social life	26
	Being consciously visually literate	20
	Realizing the importance of visuals in printed texts	17
	Visual perception skills	16
	Ability to analyze images and symbols in visuals	13
	Ability to analyze design features of visuals	10
	Ability to recognize the multi-layered structure of visuals	7
	Visual creativity and aesthetic sensitivity	5

## Data Availability

The original contributions presented in the study are included in the article; further inquiries can be directed to the corresponding author.
